# Characterizing 5-oxoproline sensing pathways of *Salmonella enterica* serovar typhimurium

**DOI:** 10.1038/s41598-022-20407-0

**Published:** 2022-09-24

**Authors:** Einav Stern, Naama Shterzer, Erez Mills

**Affiliations:** Department of Animal Sciences, Robert H. Smith Faculty of Agriculture, Food, and Environment, The Hebrew University of Jerusalem, 7610001 Rehovot, Israel

**Keywords:** Bacteriology, Bacterial physiology

## Abstract

5-Oxoproline (5OP) is a poorly researched ubiquitous natural amino acid found in all life forms. We have previously shown that *Salmonella enterica* serovar Typhimurium (*Salmonella*) responds to 5OP exposure by reducing cyclic-di-GMP levels, and resultant cellulose dependent cellular aggregation in a YfeA and BcsA dependent manner. To understand if 5OP was specifically sensed by *Salmonella* we compared the interaction of *Salmonella* with 5OP to that of the chemically similar and biologically relevant molecule, l-proline. We show that l-proline but not 5OP can be utilized by *Salmonella* as a nutrient source. We also show that 5OP but not l-proline regulates cellulose dependent cellular aggregation. These results imply that 5OP is utilized by *Salmonella* as a specific signal. However, l-proline is a 5OP aggregation inhibitor implying that while it cannot activate the aggregation pathway by itself, it can inhibit 5OP dependent activation. We then show that in a l-proline transporter knockout mutant l-proline competition remain unaffected, implying sensing of 5OP is extracellular. Last, we identify a transcriptional effect of 5OP exposure, upregulation of the *mgtCBR* operon, known to be activated during host invasion. While *mgtCBR* is known to be regulated by both low pH and l-proline starvation, we show that 5OP regulation of *mgtCBR* is indirect through changes in pH and is not dependent on the 5OP chemical structure similarity to l-proline. We also show this response to be PhoPQ dependent. We further show that the aggregation response is independent of pH modulation, PhoPQ and MgtC and that the *mgtCBR* transcriptional response is independent of YfeA and BcsA. Thus, the two responses are mediated through two independent signaling pathways. To conclude, we show *Salmonella* responds to 5OP specifically to regulate aggregation and not specifically to regulate gene expression. When and where in the *Salmonella* life cycle does 5OP sensing takes place remains an open question. Furthermore, because 5OP inhibits c-di-GMP through the activation of an external sensor, and does not require an internalization step like many studied biofilm inhibitors, 5OP or derivatives might be developed into useful biofilm inhibitors.

## Introduction

Cyclic-di-GMP (c-di-GMP) is a bacterial second messenger which in *Salmonella enterica* serovar Typhimurium (*Salmonella*), as well as other bacteria, controls a behavioral switch regulating motility versus sessility, mainly in the form of biofilm formation^1–3^. In *Salmonella*, high levels of c-di-GMP lead to protective cellulose secretion^4^. As biofilms are a major problem in medical and industrial settings, for example by forming on catheters or on gallstones, there is a need for the identification and development of biofilm inhibitors^4–6^.

*Salmonella*, like all organisms, has to sense changes in its environment in order to modify its behavior and optimize its nutrient acquisition and chances of survival. Indeed, c-di-GMP levels in bacteria are controlled by sensors which respond to extracellular cues^7^. The *Salmonella* genome encodes 17 c-di-GMP enzymes that synthesize or degrade c-di-GMP, theoretically in response to diverse environmental signals. Thus, we have previously performed a screen for nutrient type compounds that are naturally sensed by *Salmonella* to modulate the levels of c-di-GMP^8^. One of the compounds identified in our screen to negatively regulate c-di-GMP and cellulose dependent cellular aggregation, and a potential biofilm inhibitor, is 5-oxoproline (5OP).

5OP, also known as pyroglutamic acid and pidolic acid, is a ubiquitous but little studied natural amino acid derivative. It is an enzymatic intermediate in the eukaryotic γ-glutamyl cycle, converted into glutamate by 5-oxoprolinase, and is also an unavoidable damage product formed spontaneously from glutamine and other sources^9^. Furthermore, human inborn errors of metabolism that lead to 5OP buildup result in metabolic acidosis, hemolytic anemia, and neurological problems^10^. Thus, understanding if *Salmonella* indeed senses this specific molecule and characterizing how *Salmonella* responds to 5OP might shed light on the functions of this molecule. Furthermore, as *Salmonella* is a pathogen, understanding if and how 5OP sensing integrates into host–pathogen interactions might lead to new methods to prevent or treat infection.

**Figure 1:**
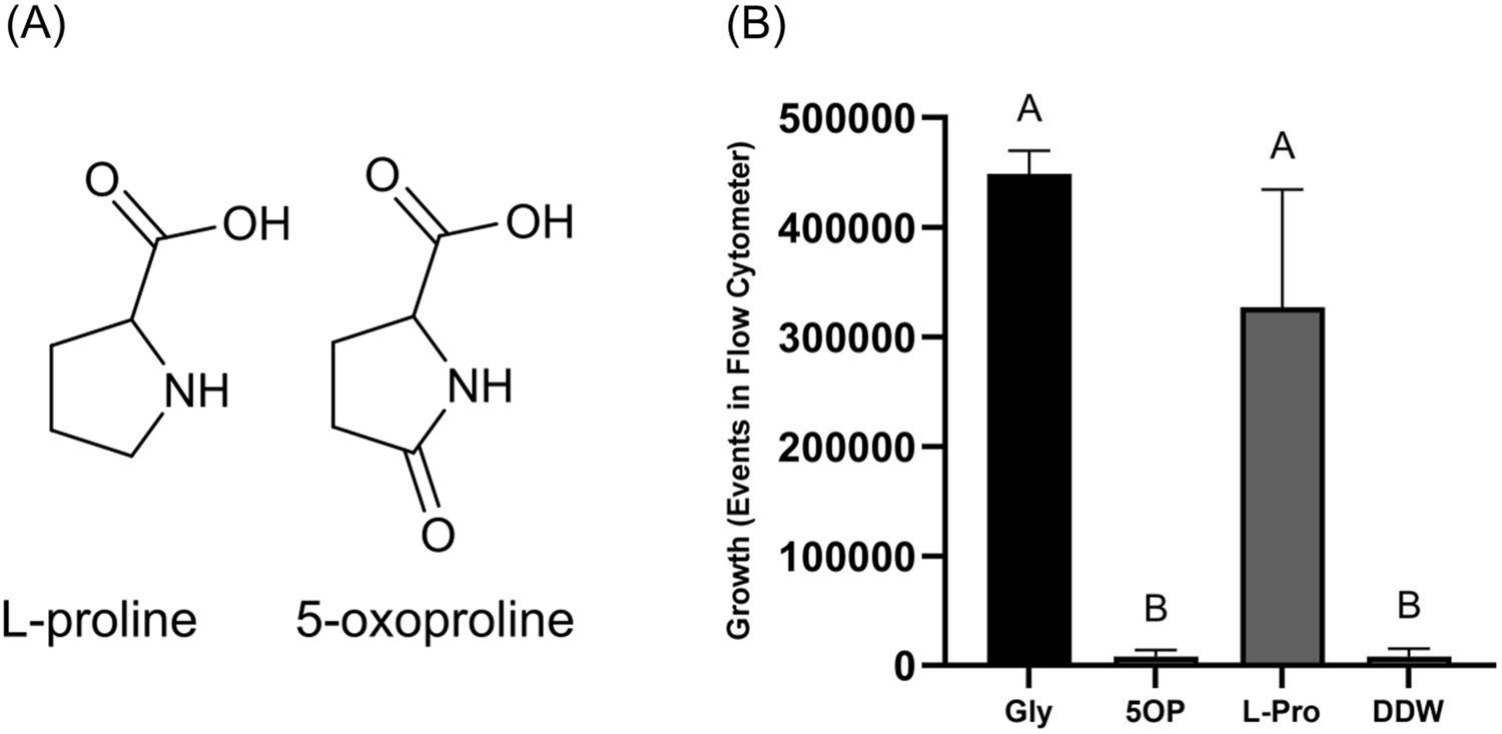
l-Proline but not 5OP can be utilized for growth. (**A**) The chemical structure of 5OP and l-proline. (**B**) Growth of wild-type *Salmonella* in minimal buffer supplemented with either 8 mM 5OP (5OP), 8 mM l-proline (l-Pro), 25 mM glycerol (Gly) as a positive control, or water as a negative control (DDW). Shown are the total number of events counted by running 5 µl of culture through a flow cytometer. Average and standard deviation of 6 biological repeats are shown. One-Way ANOVA was conducted and Geisser-Greenouse correction was used. Treatments denoted by different letters are statistically different at a P value of 0.01 or less.

Of note, l-proline is known to regulate c-di-GMP and cellulose secretion, as well as *Salmonella* virulence factors including the *mgtCBR* operon^11^ during *Salmonella* residence inside of host macrophages^12–14^. Because l-proline and 5OP are very similar molecules (Fig. 1a), we hypothesized that it is possible that the effects of 5OP are dependent on its structural similarity with l-proline. Thus, this study was aimed at characterizing 5OP sensing in *Salmonella* including its specificity and relation to l-proline sensing.

## Materials and methods

### Strain, plasmids and primers

A list of strains and plasmids can be found in Supplementary Table S1, and a list of primers used for cloning, verifying knockout strains, and for quantitative PCR (qPCR) can be found in Supplementary Table S2. Most knockout mutants were a kind gift from Prof. Roi Avraham, originally obtained from the *Salmonella enterica* serovar Typhimurium SL14028 (*Salmonella*) knockout strain library^15^. All strains derived from this library were verified by us to hold the expected mutation by sequencing the knockout area. The *putP* knockout mutant strain used in this study was generated from the same SL14028 strain background utilizing the method described by Datsenko and Wanner^16^.

### Bacterial growth conditions for flow cytometry and qPCR

Bacteria were grown for 18 h at 37 °C with shaking in 2 ml of a minimal defined medium [21 mM K_2_HPO_4_, 11 mM KH_2_PO_4_, 3.8 mM (NH_4_)_2_SO_4_, ~ 3.8 mM KOH, 25 mM glycerol, 1 mM MgCl_2_, 10 μM FeCl_3_, 1 mM NaCl, 1 × MEM amino acid solution (Gibco), 1 × MEM nonessential amino acid solution (Gibco), pH 7.4]^8^. It should be noted, that unlike the growth experiments described in the next section, in experiments analyzing aggregation through flow cytometry or RNA expression by qPCR, glycerol as a carbon source was always present. If different strains were utilized in the same experiment, OD600 was measured and bacterial concentrations were normalized. Cultures were then diluted 1:100 or 1:20 for flow cytometry or qPCR, respectively, into 5 ml of the same medium lacking both amino acid solutions, and grown for an additional 2 h at 30 °C with shaking. Cultures were then pelleted by centrifugation for 3 min at 15,000*g* speed and resuspended in new minimal media without amino acids. 5-oxoproline (5OP), l-proline, or both were diluted in water and added in a volume of 1:5 of the bacteria, to a final concentration of 8 mM for 5OP, 8 mM for l-proline, and 8 mM for 5OP and 80 mM for l-proline in competition experiments in which both were present. For flow cytometry bacteria were incubated with the compounds at 30 °C standing in 96 well plates in a total volume of 100 µl for 1 h before flow cytometry analysis. For RNA extraction, bacteria were incubated at 30 °C shaking with compounds for 2 h in a total volume of 2 ml.

### Bacterial growth measurement

Bacteria were directly inoculated from frozen stock into new minimal defined media without amino acids in which the glycerol was exchanged with 8 mM 5OP, or 8 mM l-proline. As a negative control we used minimal defined media without 5OP, l-proline, or glycerol. Bacteria were incubated for 3 days shaking at 37 °C. To measure growth, we utilized a flow cytometer (CytoFlex, Beckman Coulter Life Science company) and determined the number of events recorded by running 5 µl of culture. Supplementary Fig. S1a shows representative flow cytometer output scatter plots used for bacterial growth measurement. All events were counted. Control buffers prepared without bacteria inoculation were used to determine background events. Thus, event counts reported below are the number of events above background. To verify that flow cytometry results indeed represent live cells, we also diluted the 3 day old cultures 1:100 into LB and followed growth kinetics by OD_600_ using a plate reader. This resulted in very similar readings (Supplementary Fig. S1b) verifying flow cytometry analysis was indeed reporting growth.

### Measurement of cellular aggregation

Aggregation was measured by flow cytometry as previously described^8^. Briefly, a gate corresponding to high forward and side scatter events was established by comparing wild-type and a *bcsA* knockout mutant deficient in cellulose secretion and subsequent cellular aggregation (Supplementary Fig. S2). The same gate was used for all experiments. These high forward and side scatter events were considered aggregates. A second gate, encompassing all events above a certain threshold of forward and side scatter was established in order to count events which are likely bacterial cells but to omit events which are likely noise or buffer particles. We term this second gate “total events” and it encompasses within it the aggregate gate. The percentage of aggregate events out of the total events is reported. To make sure that high forward and side scatter events were indeed aggregates of bacteria and not a result of a change in size of single cells we imaged *Salmonella* cells by an imaging flow cytometer (image stream). We found indeed that high forward and side scatter events represented cellular aggregates (Supplementary Fig. S3).

### Measurement of *mgtCBR* expression

OD600 of each sample was recorded for quality control. Then cultures were treated at a 1:1 ratio with Protect Bacteria reagent (Qiagen) to preserve RNA. Samples were incubated at room temperature for 5 min and then pelleted by centrifugation for 10 min at 5000*g*. Supernatants were removed. Pellets were either frozen or used directly for RNA extraction. RNA was extracted using the RNeasy Protect bacteria mini kit (Qiagen) according to the manufacturer instructions. RNA quality and concentration was quantified using Nanodrop (Thermo Scientific). RNA was diluted to 50 ng/µl for cDNA reactions. cDNA was created with High-capacity cDNA Reverse Transcription Kit (Applied Biosystems) according to the manufacturer’s instructions. For qPCR, the 16S ribosomal gene was used as a housekeeping gene of reference. Primers used to quantify *mgtCBR* expression were taken from Lee and Groisman, 2012^13^. The 2(-∆∆CT) method was used for analysis.

### pH modulation

NaOH and HCl were used to modify the pH levels of cultures. NaOH was added to 5OP exposed cultures until the pH was equal to that of DDW control cultures. Similarly, HCl was added to DDW control cultures until the pH reached the levels of 5OP treated cultures. pH was quantified with pH strips (Macherey–Nagel).

### Statistics

GraphPad Prism 9.3.1 was used for statistical analysis. One-way ANOVA was performed when comparing multiple samples. T-test was conducted when just two samples were compared.

## Results

### l-Proline but not 5OP is utilized by *Salmonella* as a nutrient source

Responses to various nutrient type compounds could be an indirect response to their utilization in metabolism or a direct response to the activation of receptors that utilizes specific compounds as signals representing a certain environment. Furthermore, as 5OP is very similar chemically to l-proline, we theorized this was a good control for specificity in our assays. To determine if 5OP is utilized for growth in our experimental conditions we exchanged glycerol, found in our growth media, with either 5OP or l-proline. We found that l-proline but not 5OP was able to substitute glycerol for *Salmonella* growth (Fig. 1b). Of note, growth in 5OP was somewhat higher than the no carbon control, but this was not statistically significant and negligible compared to growth with l-proline and glycerol. To make sure this was not because 5OP was inhibiting growth, growth in a glycerol containing media with or without 5OP was compared. 5OP did not inhibit the growth of *Salmonella* (Supplementary Fig. S4). Thus, while *Salmonella* is able to efficiently internalize and utilize l-proline for growth, it is either not able to efficiently transport 5OP into the cell or not able to incorporate it into its metabolism. In any case, external 5OP is not a nutrient source *Salmonella* is able to efficiently utilize in our growth conditions. Thus, 5OP is a signal to which *Salmonella* directly responds to.

### 5OP but not l-proline regulates aggregation in a YfeA and BcsA dependent manner

**Figure 2:**
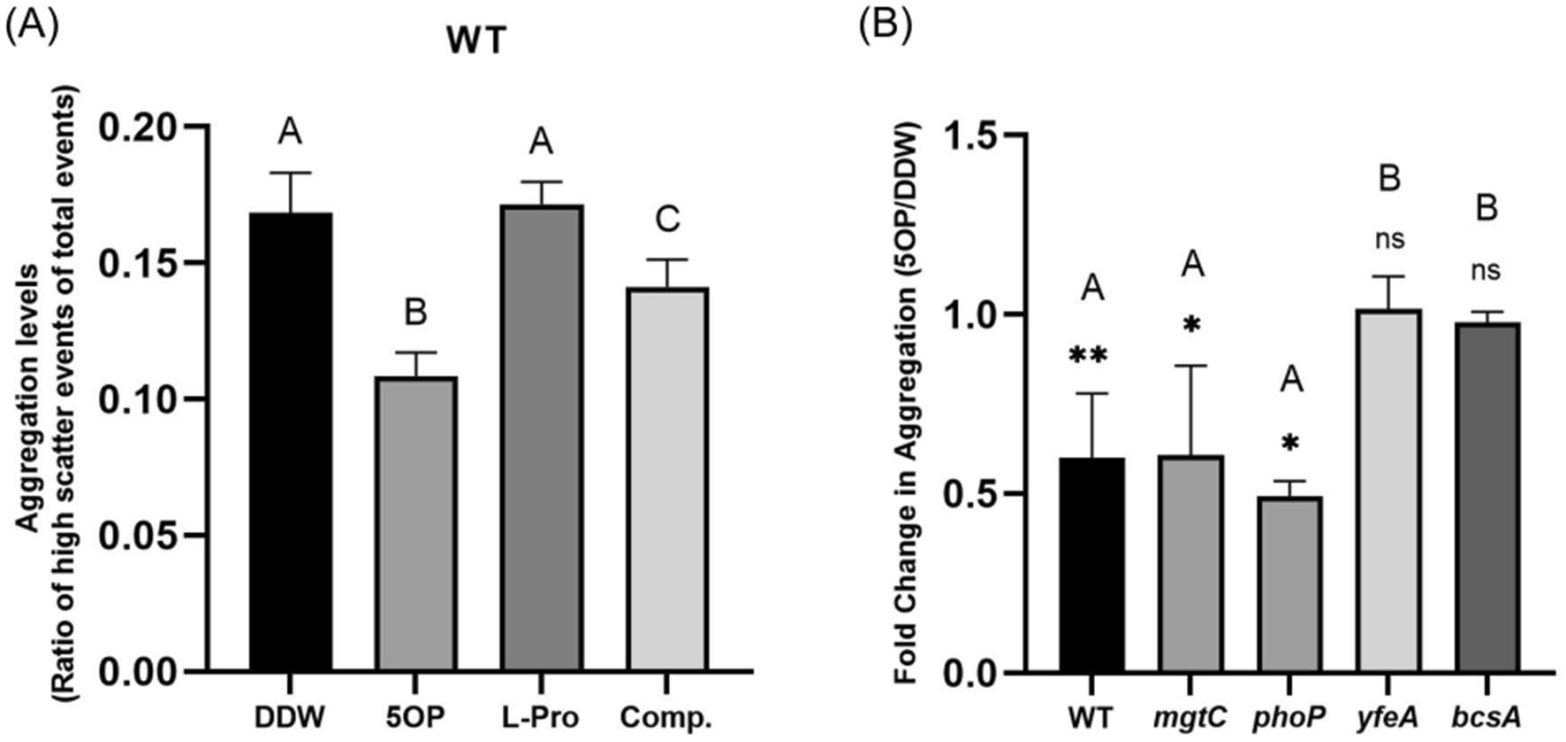
5OP but not l-proline modulates cellular aggregation in a YfeA and BcsA dependent but PhoP and MgtC independent manner. (**A**) Quantification of aggregation by flow cytometry of wild-type *Salmonella* after exposure to 5OP (5OP), l-proline (l-Pro), water (DDW), or both at a 1:10 ratio (8 mM 5OP and 80 mM l-proline, Comp.). Shown is the ratio of high light scatter events out of the total events. Results shown are the average of 5 biological repeats. One-Way ANOVA was conducted. Treatments denoted by different letters are statistically different at a P value of 0.001 or less. Standard deviation is shown. (**B**) Reduction in cellular aggregation in response to 5OP of wild-type *Salmonella* (WT), *bcsA*, *yfeA*, *phoP*, and *mgtC* knockout mutant strains. Shown is the aggregation ratio during exposure to 5OP normalized to the aggregation ratio in the DDW control. Note that wild-type, *mgtC*, *yfeA*, and *bcsA* were analyzed in one set of experiments while *phoP* was analyzed in a separate set. Shown is the average of three experiments performed. For each mutant, t-test was performed between 5OP treatment and DDW control (**P < 0.01, *P < 0.05, *ns* not significant.); One-way ANOVA was performed between fold change of different mutants. Treatments denoted by different letters are statistically different at a P value of 0.05 or less.

To examine the specificity of 5OP signaling we first confirmed and extended earlier results. As previously published^8^, exposure to 5OP resulted in a reduction in cellular aggregation (Fig. 2a). A dilution analysis showed that the aggregation phenotype is dependent on the concentration of 5OP (Supplementary Fig. S5). As expected, this phenotype was dependent on the YfeA (also called STM2410) phosphodiesterase (Fig. 2b), shown previously to be required for c-di-GMP modulation by 5OP^8^, and BcsA (Fig. 2b), a part of the cellulose secretion machinery^17^.

As l-proline is very similar structurally to 5OP (Fig. 1a) it is possible that l-proline is the molecule which is sensed rather than 5OP. To determine if this is the case we measured the ability of l-proline to modulate aggregation. However, l-proline was not able to modulate aggregation (Fig. 2a). Thus, the receptor activated by 5OP can differentiate between 5OP and l-proline.

### l-Proline is a competitor for the 5OP effect on aggregation

Because l-proline and 5OP are so similar in their chemical structure, it is possible that even though l-proline does not modulate aggregation by itself, it would compete with 5OP over binding of a hypothetical 5OP sensor. Thus, we compared the aggregation of wild type *Salmonella* exposed to just 5OP or to both 5OP and l-proline added in excess, at a 10 times higher concentration than 5OP. We found that adding l-proline in excess resulted in an attenuated aggregation response to 5OP (Fig. 2a). Thus, l-proline is an inhibitor of the 5OP aggregation response likely because of the structural resemblance between the two molecules.

### PutP is required for l-proline transport

**Figure 3:**
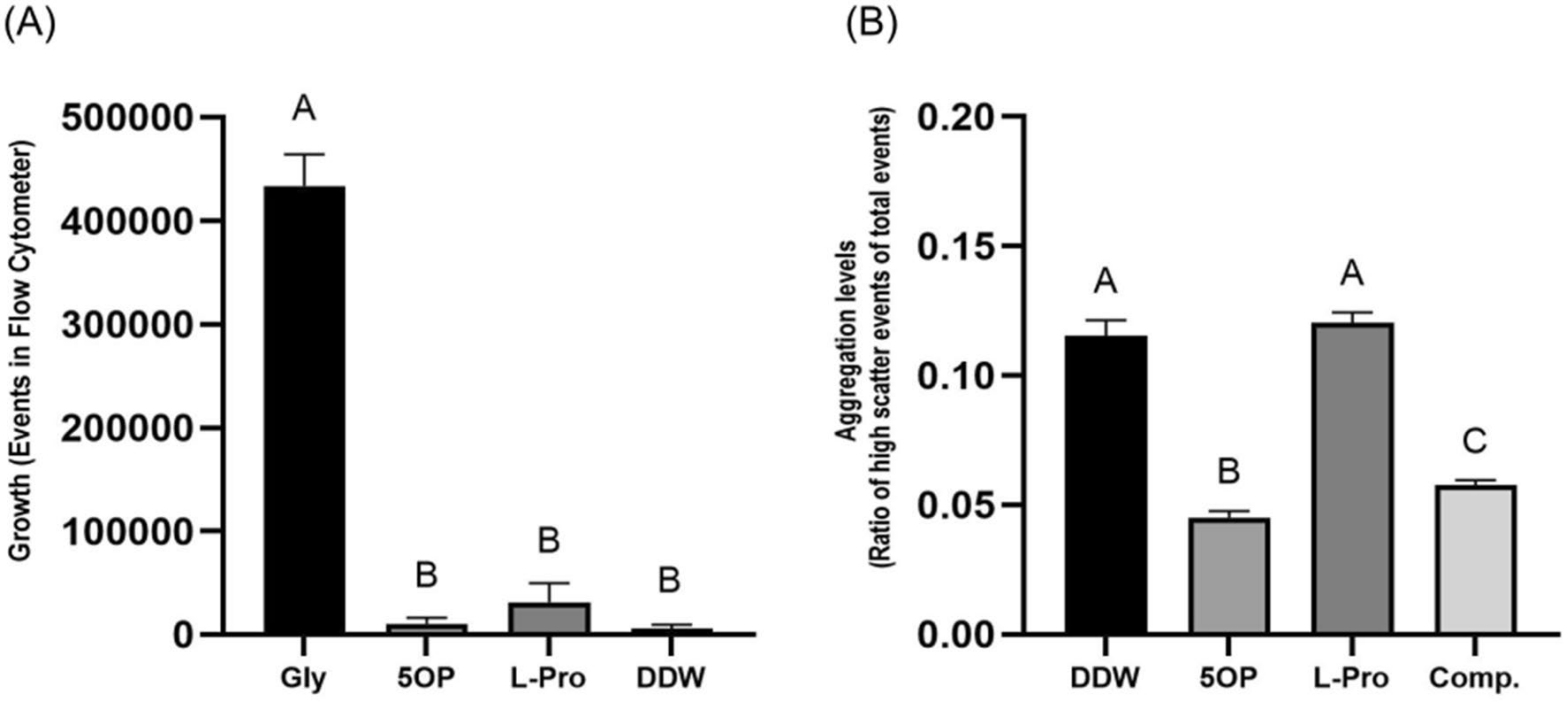
Growth and aggregation of a *putP* knockout mutant. (**A**) Growth of a *Salmonella putP* knockout mutant in minimal buffer supplemented with either 8 mM 5OP (5OP), 8 mM l-proline (l-Pro), 25 mM glycerol (Gly) as a positive control, or water as a negative control (DDW). Shown are the total number of events counted by running 5 µl of culture through a flow cytometer. Average and standard deviation of 4 biological repeats are shown. One-Way ANOVA was conducted and Geisser-Greenouse correction was used. (**B**) Quantification of aggregation by flow cytometry of the same strain after exposure to 5OP, l-proline (l-Pro), DDW, or both at a 1:10 ratio (8 mM 5OP and 80 mM l-proline, Comp.). Shown is the ratio of high light scatter events out of the total events. Due to variation between experiments a representative experiment out of three is shown in (**B**). One-Way ANOVA was conducted. Treatments denoted by different letters are statistically different at a P value of 0.01 or less for (**A**) and 0.001 or less for (**B**). Standard deviation is shown.

Because l-proline acts as an inhibitor, identifying the l-proline transporter active in our experimental conditions will be a useful tool to determine if 5OP is sensed inside the bacterial cell or externally. *Salmonella* is thought to encode at least three transporters for l-proline, the ProVWX glycine betaine/l-proline ABC system, the ProP proline/betaine transporter, and the PutP sodium/l-proline symporter^18–20^. To identify which of these is required for l-proline transport in our assay conditions, we measured the growth of the corresponding knockout mutants in a growth medium in which we exchanged glycerol with l-proline as described above. The *putP* knockout mutant completely lost the ability to grow on the l-proline based media (Fig. 3a) while the other two knockout mutants grew just as well as the wild type (Supplementary Fig. S6). Thus, in our conditions the PutP transporter was required for l-proline internalization.

### l-Proline is a competitor also in a *putP* knockout mutant background

Because l-proline likely competes with 5OP on its binding site in an unknown sensor and because PutP is required for l-proline internalization, the ability of l-proline to act as a competitor in a *putP* knockout mutant would allow to elucidate if 5OP sensing is internal or external. We found that in the *putP* knockout mutant background l-proline still functions as a competitor (Fig. 3b). Likewise, responses to 5OP and l-proline competition in *proP* and *proV* mutants were similar (Supplementary Fig. S7). Thus, 5OP is likely sensed externally by *Salmonella*.

### 5OP regulates *mgtCBR* transcription

**Figure 4:**
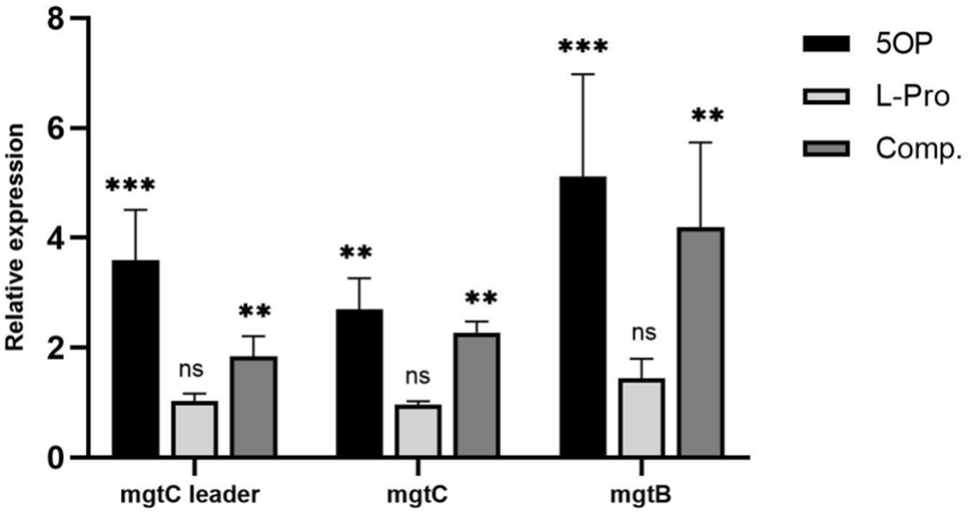
5OP upregulates the expression of the *mgtCBR* operon. Three primer pairs were used to quantify different parts of the operon. Shown is the relative expression by wild-type *Salmonella* exposed for 2 h to 5-OP (5OP), l-proline (l-Pro), or both at a 1:10 ratio (8 mM 5OP and 80 mM l-proline, Comp.) and normalized to DDW control cultures. Shown is the average of three experiments performed. One-Way ANOVA was conducted. ***P < 0.001, **P < 0.01, *ns* not significant. Geometric mean and standard error of mean are shown.

l-Proline starvation is known to regulate the expression of the pathogenicity related *mgtCBR* operon which also regulates c-di-GMP levels and cellulose secretion^12–14^. As 5OP and l-proline are very similar molecules (Fig. 1a), and because we have previously shown 5OP regulates c-di-GMP and cellulose levels^8^, we hypothesized it is possible that the mechanism of action of 5OP is to induce l-proline starvation by competitively inhibiting l-proline binding to unknown receptors or enzymes. As shown later, this hypothesis turned out to be wrong. However, this implied 5OP might also regulate *mgtCBR* transcription. Thus, we exposed *Salmonella* to 5OP, purified RNA and measured the level of transcript using three sets of primers. One set of primers targeted the mRNA leader sequence, the second set targeted *mgtC* itself, and the third set targeted *mgtB*. All three sets showed an increase in transcription in response to 5OP exposure in comparison with the unexposed control, with the *mgtB* area showing the highest increase (Fig. 4). Thus, 5OP exposure indeed regulates also *mgtCBR* as hypothesized.

### l-Proline does not regulate *mgtCBR* expression

Because our hypothesis for the 5OP mechanism of action included l-proline starvation, and because we found l-proline to be a competitor for 5OP dependent aggregation, we examined if l-proline would affect *mgtCBR* expression by itself or as a competitor to 5OP. Surprisingly, we found l-proline had no effect on *mgtCBR* expression (Fig. 4). Furthermore, because of large variation it was not clear if l-proline was a competitor for 5OP dependent *mgtCBR* regulation. To conclude, it seemed unlikely that the 5OP mechanism of action included l-proline starvation as we originally hypothesized.

### 5OP regulation of *mgtCBR* is pH and PhoPQ dependent

**Figure 5:**
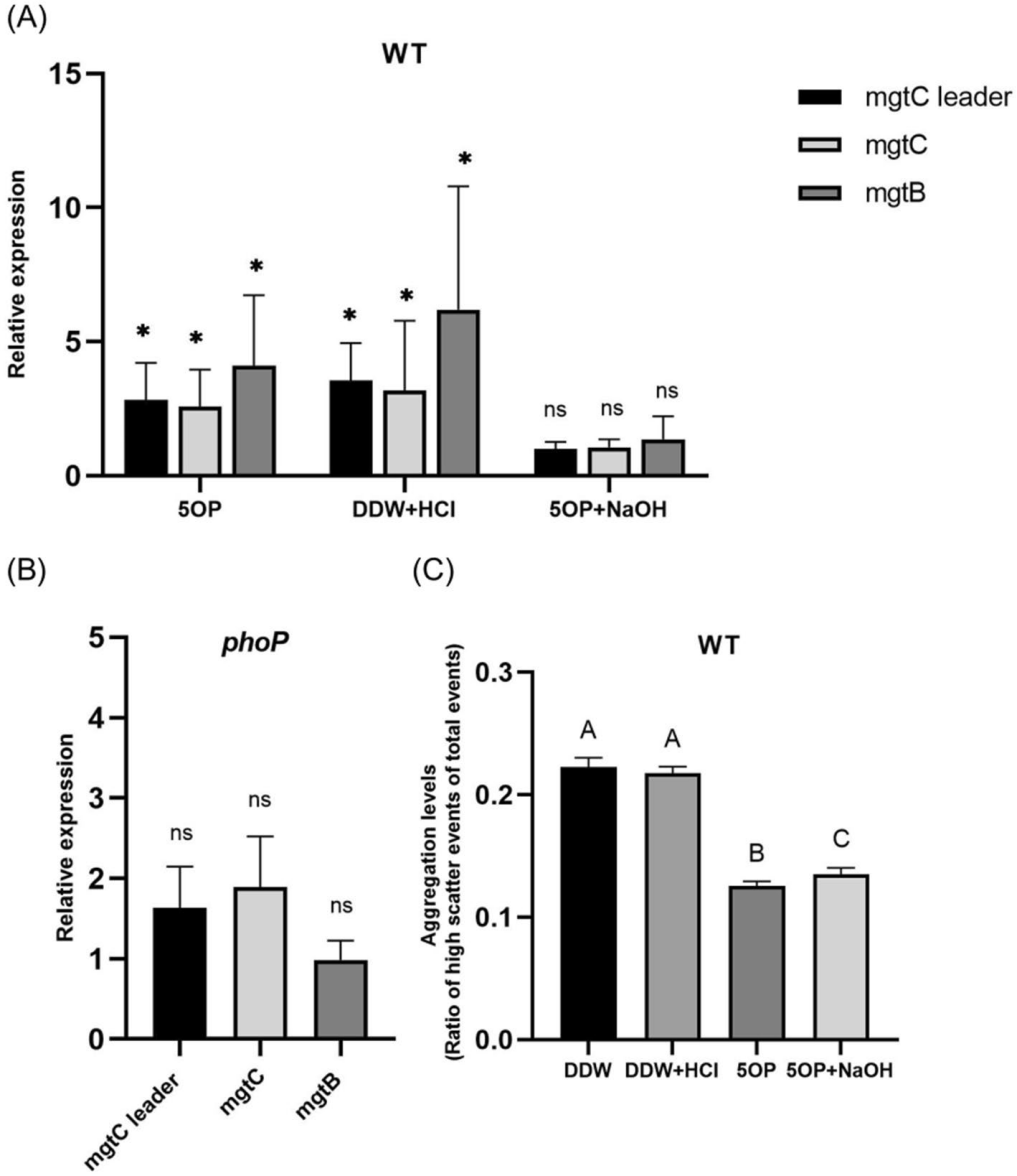
pH and PhoPQ regulate *mgtCBR* expression but not cellular aggregation. (**A**) Analysis of *mgtCBR* levels in wild-type *salmonella* exposed for 2 h to 5OP, DDW, 5OP and NaOH to restore pH (5OP + NaOH), or to DDW while pH was reduced by adding HCl (DDW + HCl). **P < 0.01, *P < 0.05, ns = not significant. (**B**) Analysis of *mgtCBR* levels in a *phoP* knockout mutant strain, exposed to 5OP or DDW. All panels represent the average of three independent experiments. One-Way ANOVA for each treatment vs. DDW control (**A**) and T-Test were conducted (**B**). ns = not significant. Geometric mean and standard error of mean are shown. (**C**) Cellular aggregation ratio of *Salmonella* wild-type exposed to 5OP, DDW, 5OP and pH restored by adding NaOH (5OP + NaOH), or to DDW while pH was reduced by adding HCl (DDW + HCl). Due to variation between experiments a representative experiment out of three is shown. One-Way ANOVA was conducted between treatments (**C**). Treatments denoted by different letters are statistically different at a P value of 0.0001 or less. Standard deviation is shown.

The *mgtCBR* operon is also known to be regulated by pH through the two component system PhoPQ^21^. Notably, 5OP (pKa = − 1.76) is a stronger acid than l-proline (pKa = 1.99). Thus, it might regulate *mgtCBR* nonspecifically by modulating pH levels. To determine if this mechanism of action is responsible for the *mgtCBR* regulation phenotype, we exposed *Salmonella* to 5OP but countered the acidity by adding NaOH. This resulted in a loss of *mgtCBR* expression (Fig. 5a). Furthermore, exposing *Salmonella* to acidic pH without 5OP produced a similar upregulation of the *mgtCBR* operon as during exposure to 5OP (Fig. 5a). Last, a *Salmonella phoP* knockout mutant did not modulate *mgtCBR* expression in response to 5OP exposure like the wild-type (Fig. 5b). Thus, *mgtCBR* regulation by 5OP was non-direct through pH modulation and not because of its similarity to l-proline.

### 5OP regulation of cellular aggregation is pH, PhoPQ and MgtC independent

The results described in the previous section show that 5OP regulates pH levels to increase *mgtCBR* operon expression through PhoPQ activation. The results also show that 5OP regulated c-di-GMP, and resultant cellulose dependent cellular aggregation through YfeA and BcsA. Because MgtC is known to regulate c-di-GMP and cellulose by itself^14^, it was possible these were all parts of one signaling pathway starting with 5OP, going through MgtC and ending with cellular aggregation. To determine if this is the case, we characterized the effect of pH on cellular aggregation: we either added NaOH to counter 5OP’s acidity, or used HCl to reduce the pH independently of 5OP. We found that even when countering 5OP’s acidity, the 5OP aggregation effect was maintained (Fig. 5c). Concurrently, changing the pH in the absence of 5OP did not result in a modulation of cellular aggregation (Fig. 5c). Furthermore, we tested if the reduction in aggregation in response to 5OP will also occur in a *phoP* knockout mutant. We found that cellular aggregation was still modulated in this mutant similarly to wild type (Fig. 2b). Thus, aggregation was independent of both pH and PhoPQ. To conclude our analysis, we also tested an *mgtC* knockout mutant. Aggregation in response to 5OP was equally reduced in a *mgtC* knockout mutant and wild type bacteria (Fig. 2b). To conclude, the cellular aggregation effect in response to 5OP is independent of the *mgtCBR* pathway.

### 5OP regulation of *mgtCBR* is independent of YfeA and BcsA

**Figure 6:**
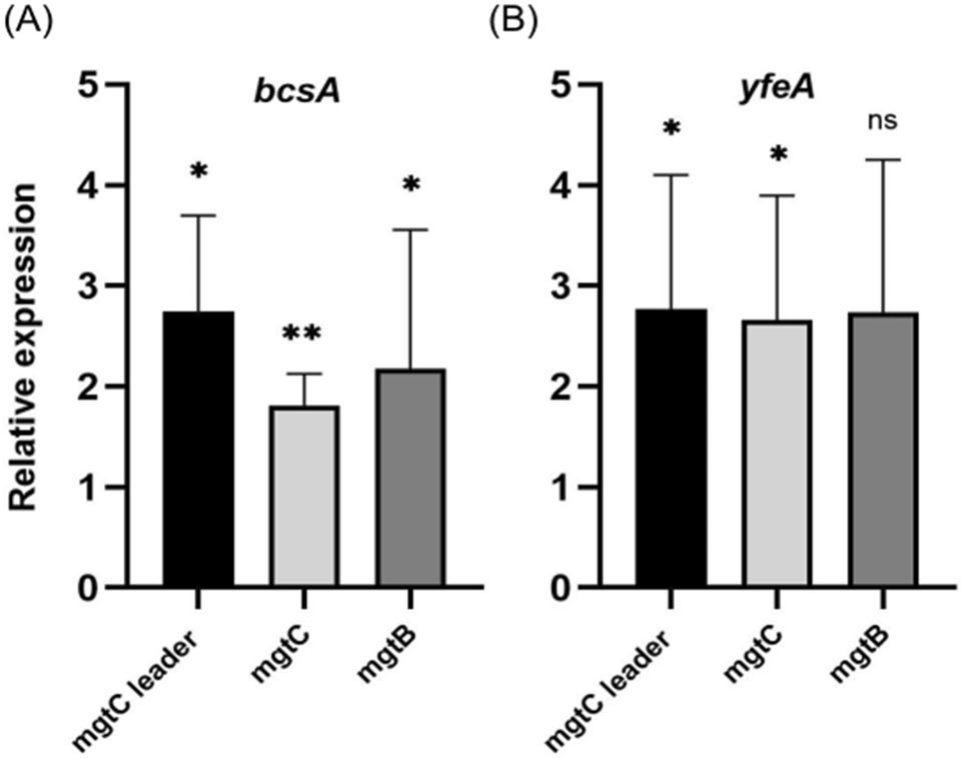
*mgtCBR* upregulation by 5OP is independent of YfeA and BcsA. Analysis of *mgtCBR* levels in a (**A**) *bcsA* or (**B**) *yfeA* knockout mutant strains, exposed to 5OP or DDW. All panels represent the average of three independent experiments. T-Test for each treatment vs. DDW control were conducted. **P < 0.01, *P < 0.05. *ns* not significant. Geometric mean and standard error of mean are shown.

While the fact that modulation of pH affected the 5OP *mgtCBR* transcriptional response but not cellular aggregation already indicated these were separate pathways, we also tested if the transcriptional response was dependent on YfeA and BcsA. We thus exposed *yfeA* and *bcsA* knockout mutants to 5OP and measured *mgtCBR* operon levels. We found that both of these mutants had increased levels of *mgtCBR* in response to 5OP just like wild type bacteria (Fig. 6a,b). Thus, 5OP dependent *mgtCBR* transcription and cellular aggregation are two independent pathways.

## Discussion

We have previously shown that 5OP is sensed by *Salmonella* to regulate c-di-GMP and cellulose dependent cellular aggregation levels^8^. Because 5OP is not a well-studied metabolite^22^, and because it is very similar to l-proline, as the two molecules differ by just one oxygen atom, it was possible that l-proline rather than 5OP was the molecule being sensed. Here we show this is not the case. 5OP rather than l-proline is sensed to regulate cellulose dependent cellular aggregation. Furthermore, we also show that modulating the pH by 5OP does not affect this specific pathway, thus the chemical structure of 5OP and not its effect on pH is sensed. Additionally, many molecules are sensed because they are nutrients utilized by the cells sensing them. In fact, at times it is difficult to determine if a molecule is actively sensed, or if physiological effects stem for the utilization of a molecule as a nutrient. Here we show that unlike l-proline, 5OP cannot be efficiently utilized as a nutrient source in our growth conditions. Finally, we show that l-proline is a competitive inhibitor for this pathway. This further proves that the specific chemical structure of 5OP is a signature sensed by a specific sensor. Thus, 5OP is specifically sensed by *Salmonella*. Presumably, it represents a specific environment for *Salmonella*. Further work identifying the environment in which 5OP is sensed is required to understand the importance of 5OP as a signal for *Salmonella*.

We also show that another result of 5OP sensing is the upregulation of the *mgtCBR* operon. As the *mgtCBR* operon is known to contribute to phagosome residence^11^, it implies a connection of 5OP sensing to pathogenicity. Furthermore, as the *mgtCBR* operon is known to be regulated by l-proline starvation^13^, it was possible that 5OP was a competitive inhibitor for l-proline binding to an unknown receptor or enzyme, possibly emulating l-proline starvation. However, *mgtCBR* is also regulated by pH^21^. Indeed, we found that it was modulation of pH and not l-proline starvation by 5OP which resulted in upregulation of the *mgtCBR* operon. Thus, regulation of *mgtCBR* transcription by 5OP, unlike the c-di-GMP/cellulose sensing pathway, is nonspecific and dependent on changes in pH levels rather than on the specific chemical structure of 5OP.

Because we have shown 5OP to regulate c-di-GMP and cellulose dependent cellular aggregation^8^ and because here we show that 5OP regulates the *mgtCBR* operon, and finally because this operon is known to regulate c-di-GMP levels and cellulose^14^, we initially theorized that these two pathways were connected. According to this hypothesis, 5OP would signal through pH modulation to activate PhoPQ, upregulating the *mgtCBR* operon, which would then regulate c-di-GMP, cellulose and cellular aggregation. However, multiple results showed this was not the case. *mgtCBR* regulation, but not aggregation, depended on the two-component system PhoPQ. Inversely, aggregation, but not *mgtCBR* regulation, depended on YfeA and BcsA. Moreover, the aggregation pathway was also independent of MgtC itself. Thus, these two pathways are completely separate. Furthermore, because *mgtCBR* regulation, but not aggregation, depended on pH modulation rather than on the specific chemical structure of 5OP, these are two separate signaling pathways already at the sensor level.

The fact that the aggregation pathway is independent of MgtC is somewhat surprising, as previous work showed that MgtC regulates c-di-GMP and cellulose through a pathway requiring ATP synthase^14^. However, an important difference in the two described pathways is that the MgtC pathway described is indirect, relaying on long term changes in ATP levels, with phenotypes identified 6 h into growth. Likely, our experiments were too short to identify the described effects of a *mgtC* knockout mutant. Moreover, it is important to note that *Salmonella* encodes 17 c-di-GMP modulating enzymes, further emphasizing the fact that many signaling pathways in *Salmonella* regulate c-di-GMP, cellulose secretion, and cellular aggregation.

Sensing can be of the internal environment or the external environment. Indeed, *Salmonella* senses both internal l-arginine levels through ArgR^23^ and external l-arginine levels through ArtI^8^. Here we show 5OP regulates *mgtCBR* expression through modulation of pH levels utilizing the PhoPQ two component system. Thus, *mgtCBR* is modulated by external 5OP. To determine if 5OP dependent aggregation was controlled by internal or external 5OP we utilized the *putP* mutant, deficient in l-proline import. We show l-proline continues to ameliorate 5OP aggregation also in the *putP* knockout mutant, which we have shown does not utilize l-proline as a nutrient source likely because l-proline is not internalized. Therefore, 5OP is sensed externally and not internally.

As discussed above, PhoPQ is the sensor system responding to 5OP pH modulation to regulate *mgtCBR*. The 5OP sensor which regulates c-di-GMP, cellulose secretion, and cellular aggregation is likely YfeA itself. YfeA includes an EAL cytoplasmic domain with a phosphodiesterase activity as well as a seven transmembrane domain termed MASE1 which is its sensor domain. Thus, the external parts of YfeA’s MASE1 domain either bind 5OP directly or indirectly. We have previously shown that in the case of l-arginine, the direct sensor was a periplasmic l-arginine binding protein, ArtI, which activates the diguanylate cyclase, YedQ (also called STM1987). Thus, it is also possible that YfeA is not the direct 5OP sensor, but that another protein, upstream in the signaling cascade, binds 5OP. One possibility for such a protein is ProX, the substrate binding periplasmic protein of the ProVWX glycine betaine ABC transporter, which in *Escherichia coli* is known to bind l-proline^24^. However, numerous additional substrate binding periplasmic proteins are encoded by *Salmonella* with unknown substrate specificities.

**Figure 7:**
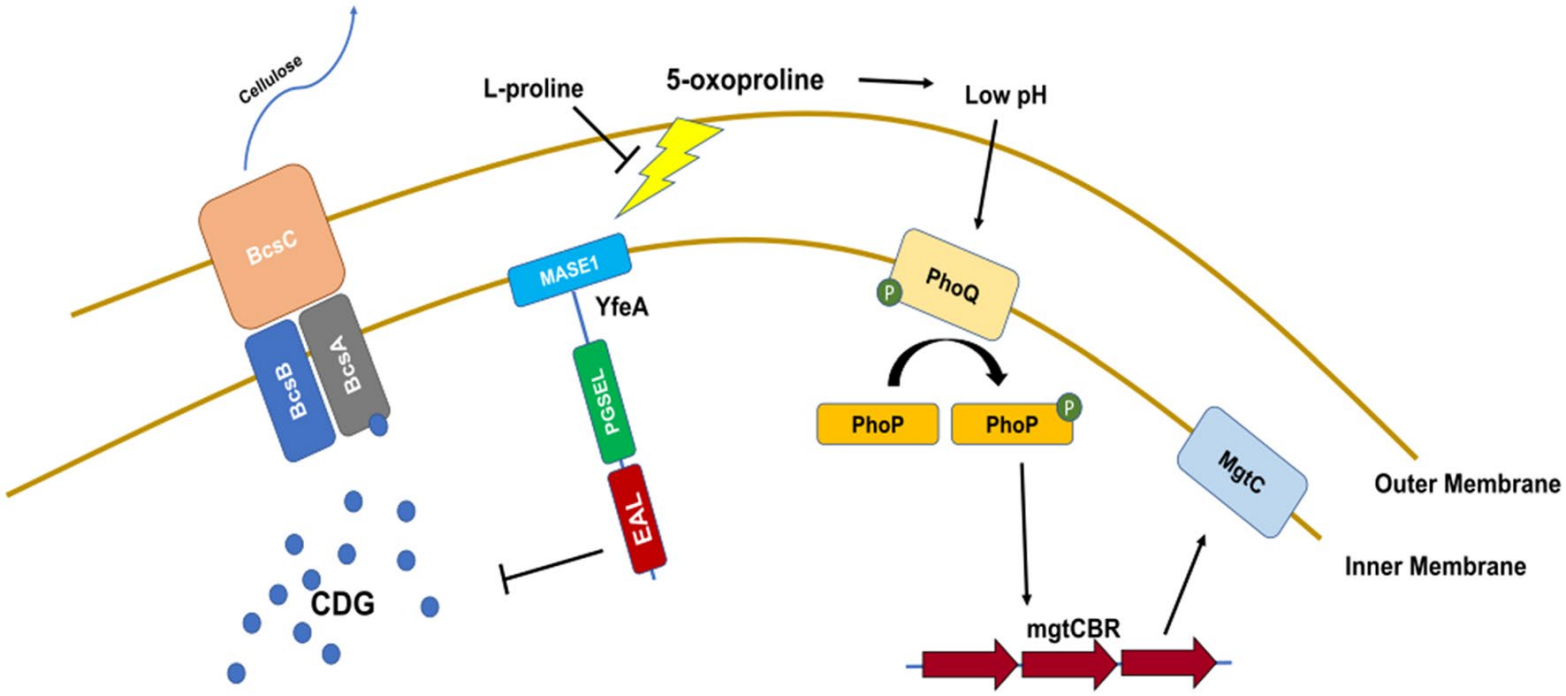
Model of 5OP sensing by *Salmonella*. Shown are the two suggested pathways. A pathway which responds specifically to 5OP, in which YfeA is activated to reduce c-di-GMP levels, thereby reducing cellulose secretion by the BCS machinery, resulting in reduced cellular aggregation. A second pathway is activated by 5OP in a non-specific manner by the reduction in pH levels. In this pathway, PhoPQ is activated to upregulate *mgtCBR* operon expression.

To conclude, in this work we show that 5OP sensing has two effects on *Salmonella* (Fig. 7). The first is a non-direct effect through modulation of pH on the expression of the *mgtCBR* operon through PhoPQ. The second effect is the result of specific sensing of 5OP through the activation of the YfeA phosphodiesterase to reduce c-di-GMP levels. While we quantified cellular aggregation, a second phenotype, harder to characterize at short time scales, is YcgR dependent regulation of flagella based swimming^25–27^. Thus, while in our laboratory conditions we measured BcsA dependent cellulose secretion and subsequent cellular aggregation, the relevant physiological phenotype may be motility, which is enhanced in low c-di-GMP levels. Only upon identification of the relevant environment in which *Salmonella* senses 5OP it would be possible to conclude which of the possible phenotypes, *mgtCBR* expression, cellular aggregation or motility is the natural target of 5OP sensing.

## Supplementary Information

Supplementary Information.

## Data Availability

All data generated or analysed during this study are included in this published article and its supplementary information file.
