# Supplementary material for: Characterizing 5-oxoproline sensing pathways of Salmonella enterica serovar typhimurium
Source: Sci Rep. 2022 Sep 24;12:15975. doi: 10.1038/s41598-022-20407-0 (PMC9509341; doi:10.1038/s41598-022-20407-0)
Supplement: Supplementary file 1 — Supplementary Information. [file 41598_2022_20407_MOESM1_ESM.docx]

**Title:**

Characterizing 5-oxoproline sensing pathways of *Salmonella* *enterica* serovar Typhimurium

**Running title:** *Salmonella* 5-oxoproline sensing

Einav Stern, Naama Shterzer and Erez Mills*

Department of Animal Sciences, Robert H. Smith Faculty of Agriculture, Food, and Environment, The Hebrew University of Jerusalem, Rehovot 7610001, Israel

*Corresponding author:

Erez Mills

Phone number: +972-08-9489576

Email: erez.mills@mail.huji.ac.il

**
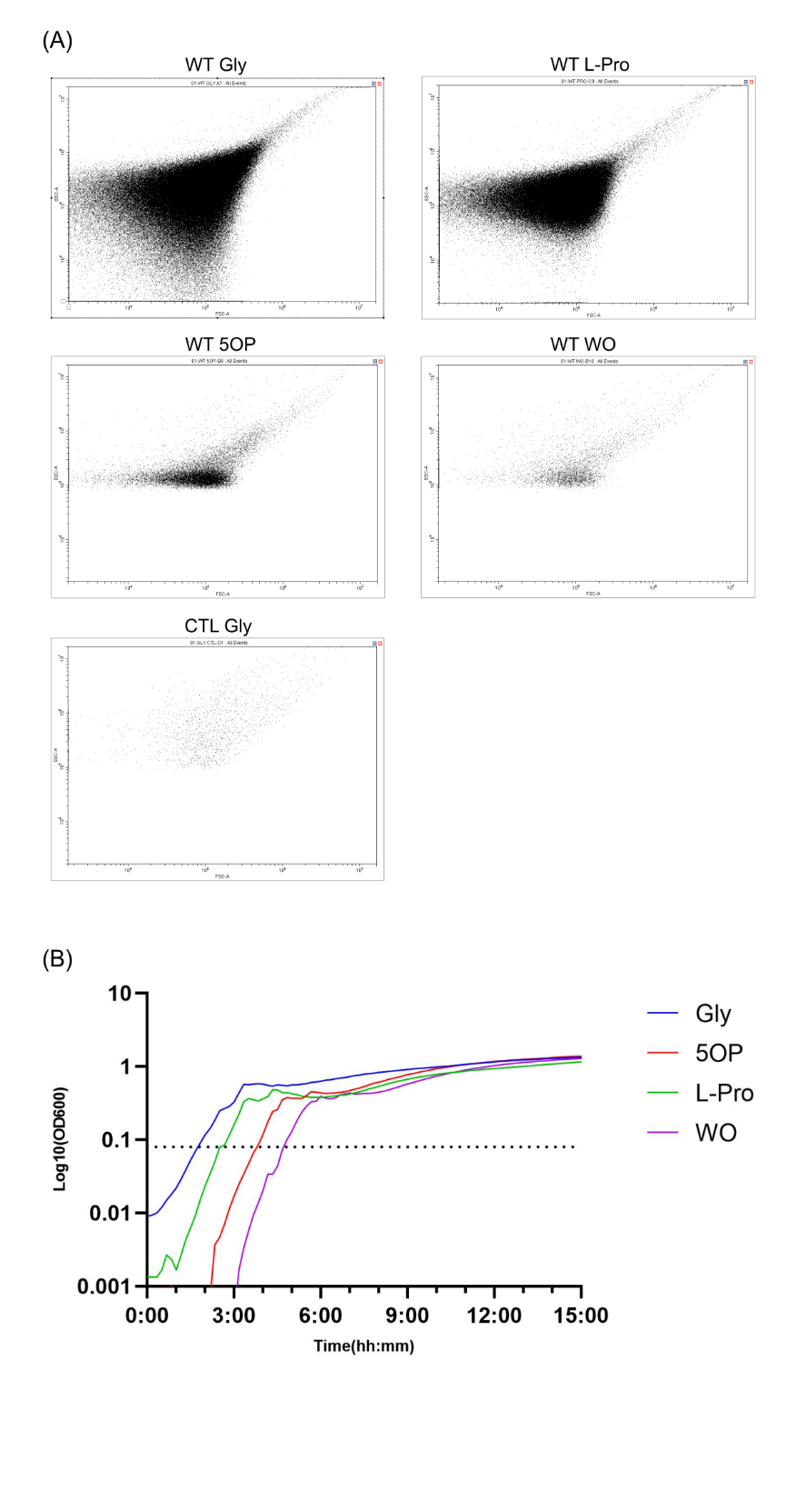
Supplementary Figure 1:** (A) Example scatter plots for growth analysis by flow cytometry. *Salmonella* wild-type (WT) was grown for three days in minimal buffer with either glycerol (Gly), L-proline (L-Pro), 5-oxoproline (5OP), or no carbon source (WO) as a control. Another control shown is minimal buffer with glycerol but no bacteria (CTL Gly). Note that for growth analysis all events were counted. To remove background events, buffer only controls were also analyzed and used for normalization of data in the main figures. (B) Analysis of survival after three-day growth. Samples shown in (A) at the end of the three-day growth period were diluted 1:100 into LB and OD600 was measured over time. Glycerol – Gly, 5-oxoproline – 5OP, L-proline – L-Pro, no carbon source during the three-day growth – WO.

**
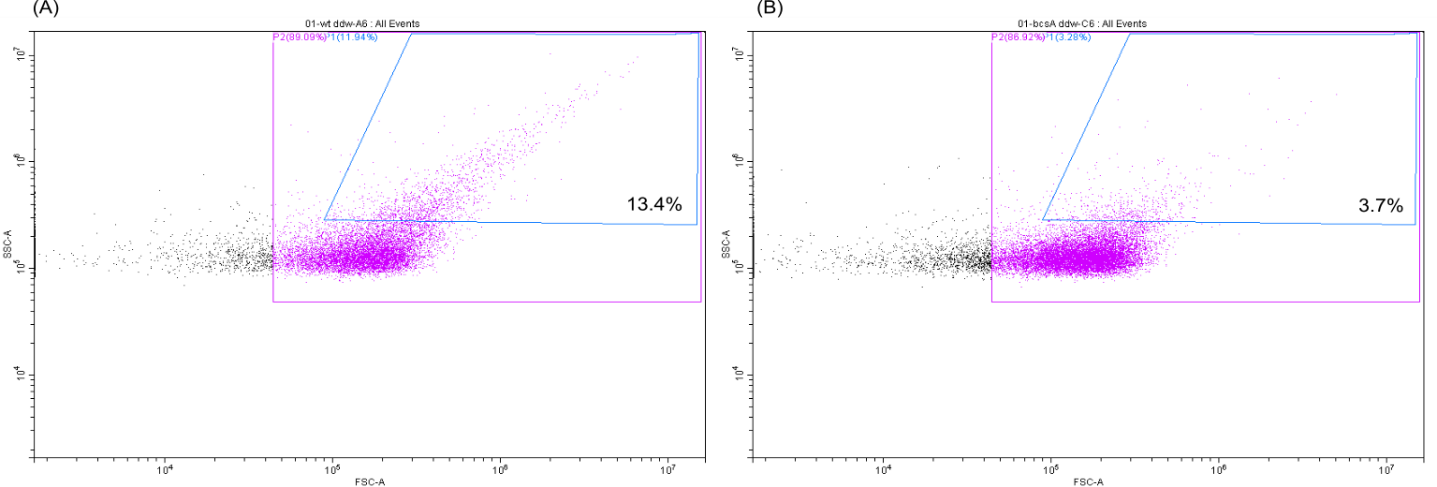
**

**Supplementary Figure 2:** Gating for the quantification of cellular aggregation. Shown are flow cytometer output graphs showing forward and side scatter. (A) Wild-type *Salmonella*. (B) *bcsA* knockout mutant. Shown is a general gate designed to exclude noise and an inner gate designed to include aggregates. The ratio in the number of events recorded in the inner gate compared to the number recorded by the outer gate is used for cellular aggregation quantification. One representative experiment is shown for both panels.


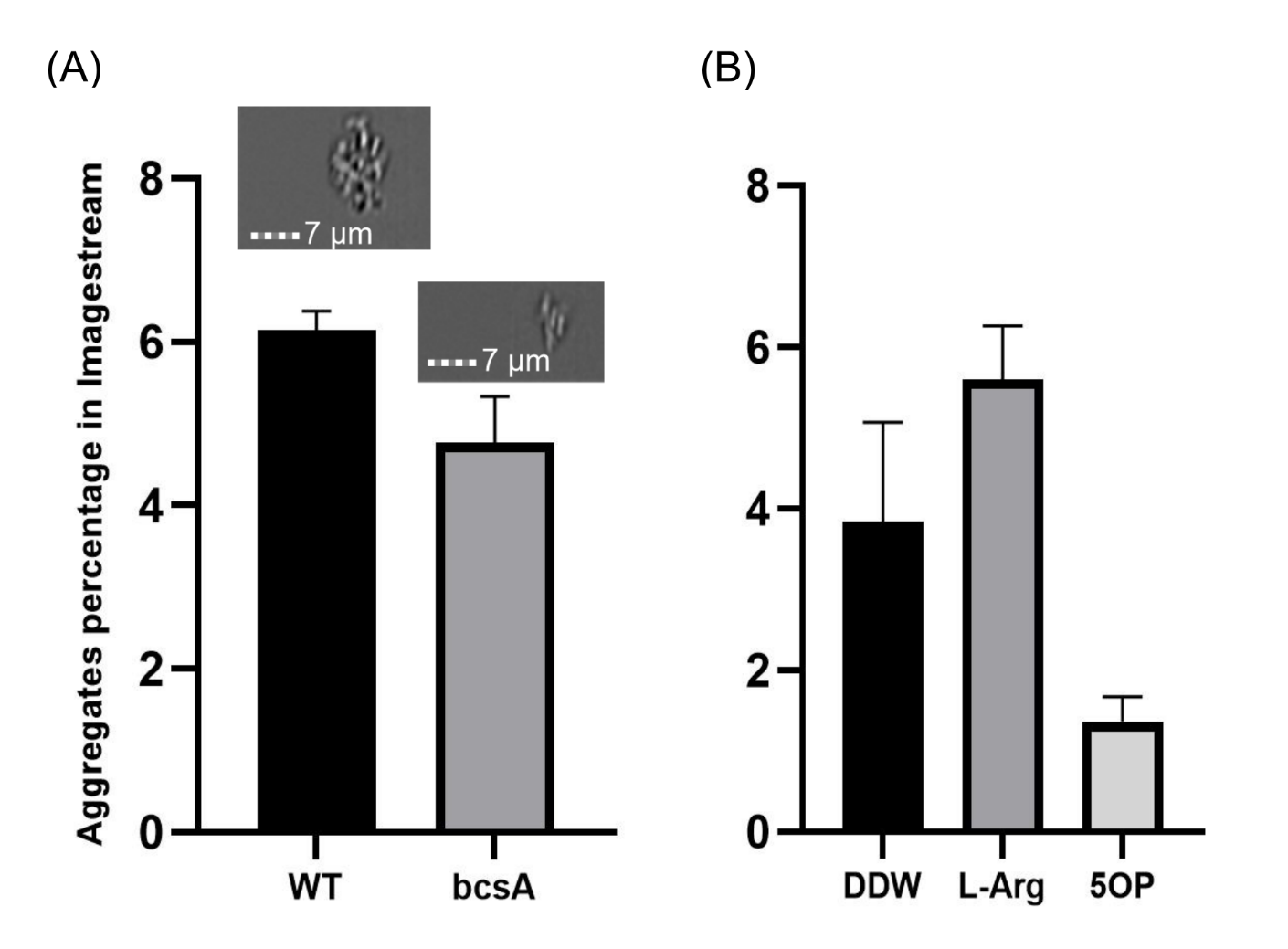


**Supplementary Figure 3:** Quantification of aggregation by an imaging flow cytometer. Cells were acquired by an ImageStreamX mark II flow cytometer (Amnis Corporation, part of

Luminex, Austin, TX), using a 60x lens (NA=0.9). Data was analyzed using IDEAS 6.3 software

(Amnis Corporation, WA, USA). Cells were gated for focused cells using the Gradient RMS

feature (measures the sharpness quality of an image by detecting large changes of pixel values in

the image, computed using the average gradient of a pixel normalized for variations in intensity

levels). Single cells and aggregates were identified by using the Area (sum of pixels within a

mask, in square microns) and Aspect Ratio (the Minor Axis divided by the Major Axis), done on

the OBJECT mask (segments images to closely identify the area corresponding to the cell. The

mask characterizes the background pixels using a set of features and then segments the image by

determining all the pixels that deviate from the background feature set). Additional aggregates

were identified by using a custom-made classifier (based mainly on various texture features), and

added to the analysis as aggregates. (A) Percent of events which were larger than a doublet of cells. Shown is an analysis of wild-type (WT) *Salmonella* as well as a *bcsA* knockout mutant (bcsA). Also shown are representative images of the largest events for each sample. (B) Quantification as above of wild-type *Salmonella* exposed to L-arginine (L-Arg) to induce aggregation, 5-oxoproline (5OP) to inhibit aggregation, or DDW as a control.


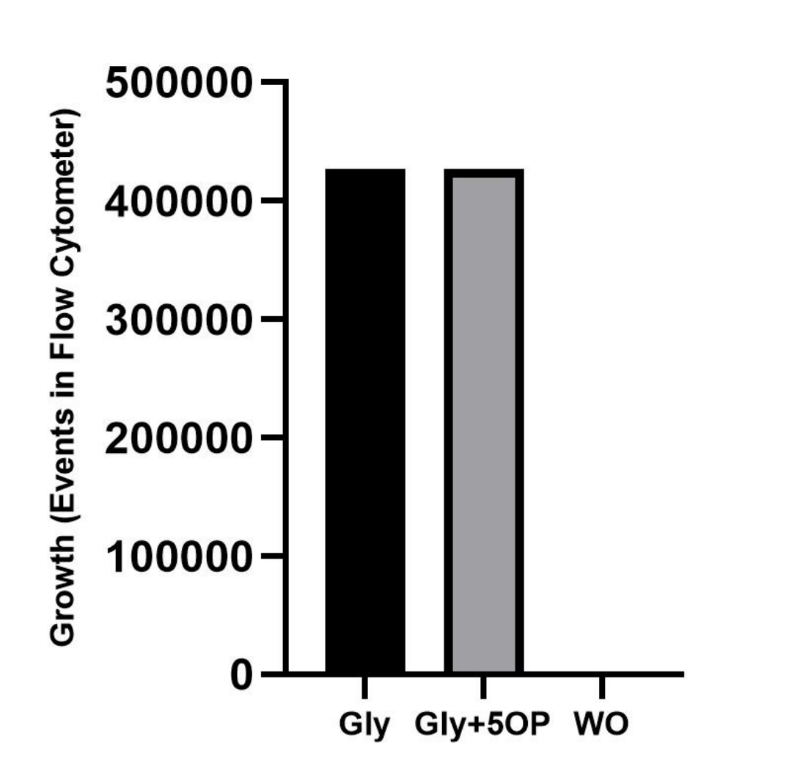
**Supplementary Figure 4:** 5-oxoproline does not inhibit *Salmonella* growth. *Salmonella* wild-type was grown in minimal media with glycerol as a carbon source with (Gly+5OP) or without (Gly) 8mM 5-oxoproline. A no carbon source control was also included (WO).


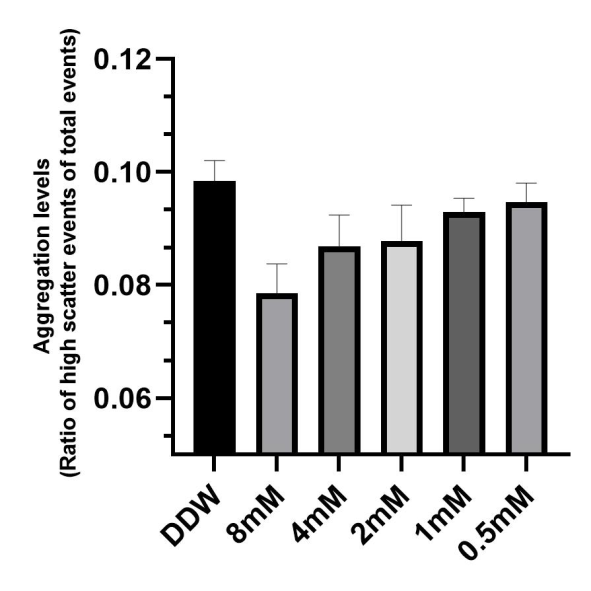
**Supplementary Figure 5:** Aggregation is dependent on the concentration of 5OP. *Salmonella* wild-type was exposed to increasing concentrations of 5OP and aggregation was determined by flow cytometry.

**
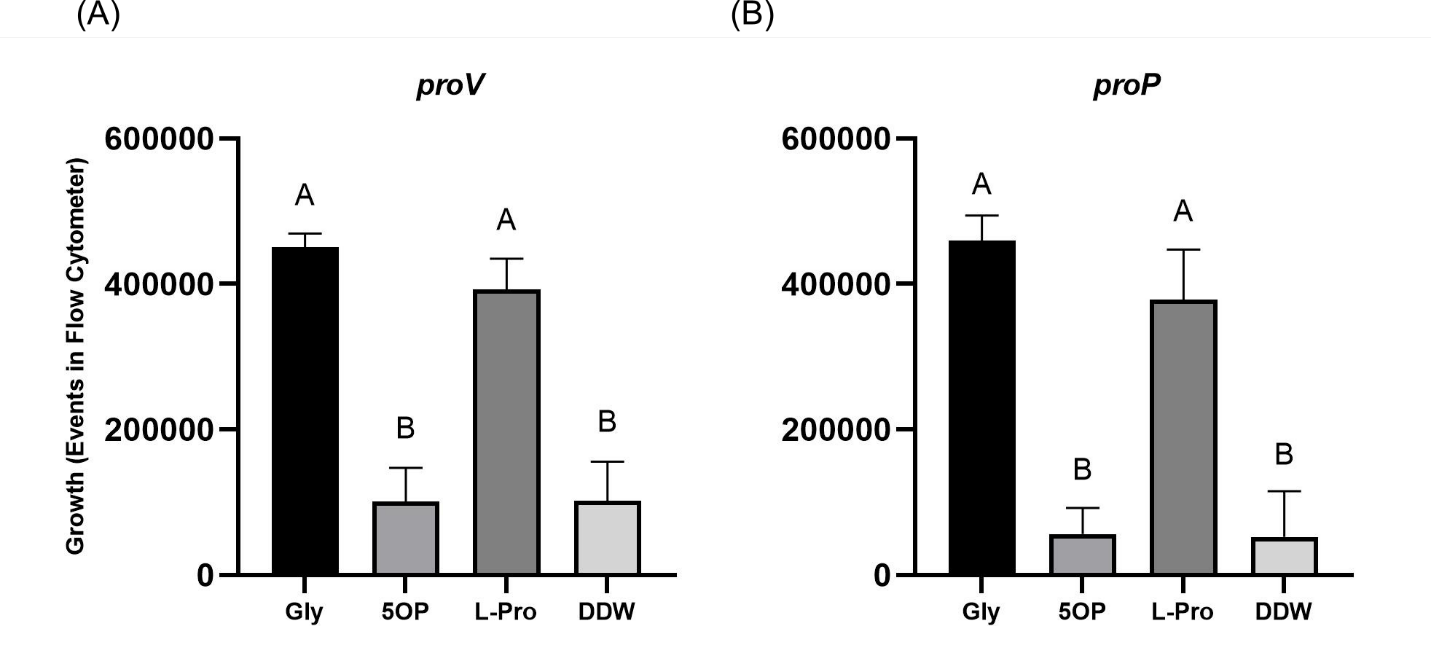
**

**Supplementary Figure 6:** L-proline utilization for growth does not depend on the ProV or ProP transporters. (A) Growth of *proV* or (C) a *proP* knockout mutant in minimal buffer supplemented with either 8mM 5OP, 8mM L-proline, 25mM glycerol as a positive control, or DDW as a negative control. Shown are the total number of events counted by running 5 µl of culture through a flow cytometer. Each panel is the average of three experiments performed. One-Way ANOVA was conducted. Treatments denoted by different letters are statistically different at a P value of 0.05 or less. Standard deviation is shown.

**
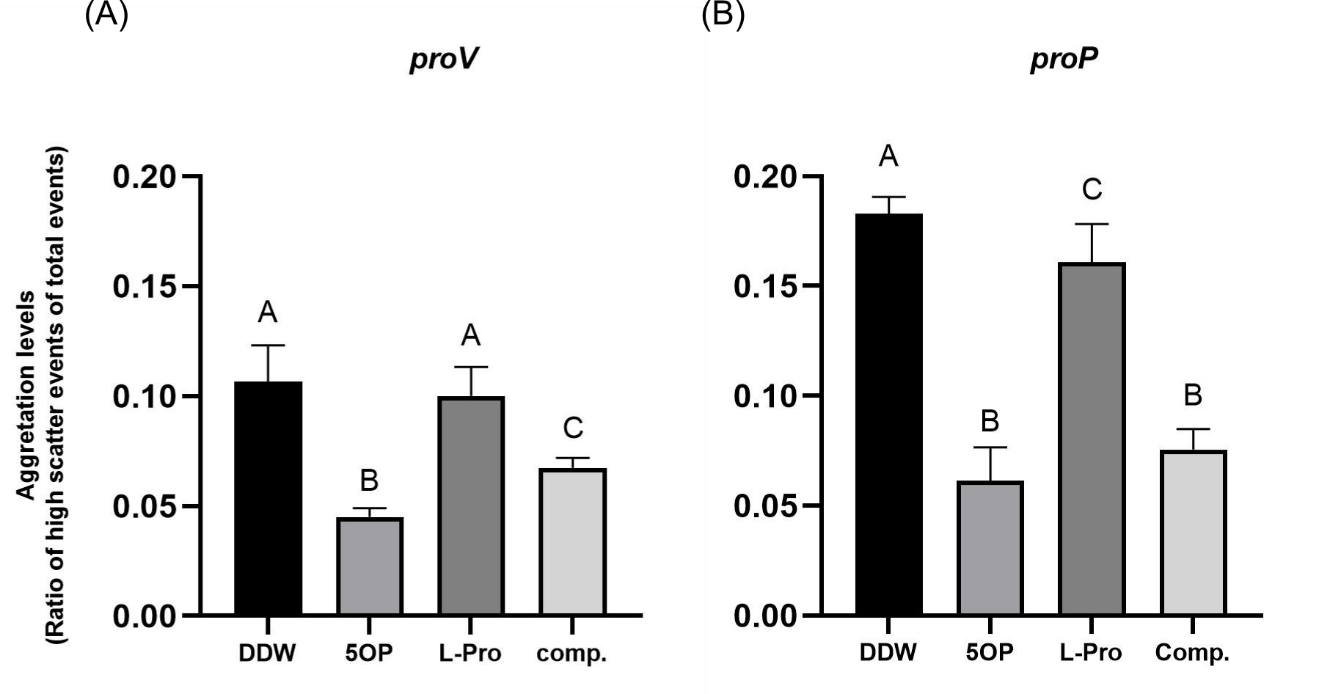
**

**Supplementary Figure 7:** Response to 5OP and L-proline competition do not require ProP or ProV. Quantification of aggregation by flow cytometry of *proV* (A), or *proP* (B) knockout mutants, after exposure to 5-OP, L-proline, DDW, or both at a 1:10 ratio (8 mM 5OP and 80 mM L-proline). Shown is the ratio of high light scatter events out of the total events. One-Way ANOVA was conducted. Treatments denoted by different letters are statistically different at a P value of 0.05 or less. Due to variation between experiments a representative experiment out of three is shown for both panels. Average and standard deviation is shown.

**Supplementary Table 1:** Strains and plasmids used in this study.

| **Bacterial/plasmid name** | **Reference** |
| --- | --- |
| wild-type | (Mills et al. 2015) |
| *∆bcsA::kan* | (Mills et al. 2015) |
| *∆yfeA::kan* | (Mills et al. 2015) |
| *∆mgtC::kan* | (Porwollik et al.) |
| *∆prop::kan* | (Porwollik et al.) |
| *∆proV::kan* | (Porwollik et al.) |
| *∆ybgK::kan* | (Porwollik et al.) |
| *∆phoQ::kan* | (Porwollik et al.) |
| *∆putP::Cam* | Created during this research |
| SL14028 pkD46(Amp^R^) | (Mills et al. 2015) |
| pKD3 | (Datsenko and Wanner 2000) |
| pKD4 | (Datsenko and Wanner 2000) |
| pKD46 | (Doublet et al.) |

**Supplementary Table 2:** Primers used for creating the *putP* knockout mutant, verifying mutations of gifted knockout strains, or qPCR.

| **No. of primer** | **Name of primer** | **Reaction** | **Sequence (5'-3')** | **Reference** |
| --- | --- | --- | --- | --- |
| 1 | mgtC leader_F | qPCR | GGGAGATTGCTGCCCACC | (Lee and Groisman 2012) |
| 2 | mgtC leader_R | qPCR | GCAGGAGTAATATGTTGGACAGTCAC | (Lee and Groisman 2012) |
| 3 | mgtC_F | qPCR | TTGTCTCTGGGATTGGCTTTCT | (Lee and Groisman 2012) |
| 4 | mgtC_R | qPCR | CAGCCCGCGCACATTC | (Lee and Groisman 2012) |
| 5 | mgtB_F | qPCR | CAGAAAATGATAAGCAGCATAAAAAA | (Lee and Groisman 2012) |
| 6 | mgtB_R | qPCR | CCCTGACGATGGCTGTTCA | (Lee and Groisman 2012) |
| 7 | HDA_F | qPCR | ACTCCTACGGGAGGCAGCAGT | (Walter et al. 2000) |
| 8 | HDA_R | qPCR | GTATTACCGCGGCTGCTGGCAC | (Walter et al. 2000) |
| 9 | mgtC_F | PCR | CCTCCG CCGTTAACAC GACG | Created during this research |
| 10 | mgtC_R | PCR | CAC GATTAGAAGG TGAATGC | Created during this research |
| 11 | proP_F | PCR | GGACAGT GTAAGTAAAC CTG | Created during this research |
| 12 | proP_R | PCR | CGTTT AATACGTCGT GACCC | Created during this research |
| 13 | proV_F | PCR | CCTCGCAATA TTCATGCCAG | Created during this research |
| 14 | proV_R | PCR | GAAAAGTGTT CAGGGGCTGG | Created during this research |
| 15 | putP_F | PCR | CACCG CTTCCGGCAG GATAC | Created during this research |
| 16 | putP_R | PCR | GGTCCGG GACTTATCAG AGG | Created during this research |
| 17 | putP_AF | Mutant preparation | tgGCTATTAGCACACCGATGTTGGTGACATTCTGTGTCTATATTTTTGGCgtgtaggctggagctgcttc | Created during this research |
| 18 | putP_AR | Mutant preparation | taTTCCGCCTGTAGCTTCGACGGCGGCGCGGAATGATAATGCGCGTCCGCcatatgaatatcctccttag | Created during this research |
